# Mechanical Enhancement of Cytocompatible 3D Scaffolds, Consisting of Hydroxyapatite Nanocrystals and Natural Biomolecules, Through Physical Cross-Linking

**DOI:** 10.3390/bioengineering7030096

**Published:** 2020-08-19

**Authors:** Despoina Brasinika, Elias P. Koumoulos, Kyriaki Kyriakidou, Eleni Gkartzou, Maria Kritikou, Ioannis K. Karoussis, Costas A. Charitidis

**Affiliations:** 1 BioG3D–New 3D printing technologies, 1 Lavriou Str., Technological & Cultural Park of Lavrion, 19500 Lavrion, Greece; dbras@biog3d.gr; 2 School of Chemical Engineering, National Technical University of Athens, 9 Iroon Polytechniou Str., Zografou Campus, 15780 Athens, Greece; elikoum@chemeng.ntua.gr (E.P.K.); egartzou@chemeng.ntua.gr (E.G.); mkritikou@chemeng.ntua.gr (M.K.); 3 School of Dentistry, National and Kapodistrian University of Athens, 2 Thivon Str., Goudi, 11527 Athens, Greece; kyriakidou_it@yahoo.it (K.K.); ikaroussis@dent.uoa.gr (I.K.K.)

**Keywords:** bone regeneration, 3D scaffolds, mechanical properties, physical cross-linking, riboflavin

## Abstract

Bioinspired scaffolds mimicking natural bone-tissue properties holds great promise in tissue engineering applications towards bone regeneration. Within this work, a way to reinforce mechanical behavior of bioinspired bone scaffolds was examined by applying a physical crosslinking method. Scaffolds consisted of hydroxyapatite nanocrystals, biomimetically synthesized in the presence of collagen and l-arginine. Scaffolds were characterized by X-ray diffraction, Fourier transform infrared spectroscopy, scanning electron microscopy (SEM), microcomputed tomography, and nanoindentation. Results revealed scaffolds with bone-like nanostructure and composition, thus an inherent enhanced cytocompatibility. Evaluation of porosity proved the development of interconnected porous network with bimodal pore size distribution. Mechanical reinforcement was achieved through physical crosslinking with riboflavin irradiation, and nanoindentation tests indicated that within the experimental conditions of 45% humidity and 37 °C, photo-crosslinking led to an increase in the scaffold’s mechanical properties. Elastic modulus and hardness were augmented, and specifically elastic modulus values were doubled, approaching equivalent values of trabecular bone. Cytocompatibility of the scaffolds was assessed using MG63 human osteosarcoma cells. Cell viability was evaluated by double staining and MTT assay, while attachment and morphology were investigated by SEM. The results suggested that scaffolds provided a cell friendly environment with high levels of viability, thus supporting cell attachment, spreading and proliferation.

## 1. Introduction

The major role of native bones is to provide structural framing and mechanical strength and support to the body. Bones typically possess the ability of self-healing [1]; however; clinical restoration is deemed necessary in case of extended injuries, as in case of car incidents, injuries and/or bone cancer removal. In bone tissue engineering approaches, one of the main challenges underpinning the development of functional scaffolds for treating difficult segmental and contained skeletal defects, refers to the mimicking of biological, structural, and mechanical characteristics of natural tissue [2,3]. 

Indeed, as a prerequisite, engineered scaffolds should be biocompatible and biodegradable, thus initiating negligible immune response upon implantation, while promoting cells interaction and bioactivity. Towards this direction, the combination of natural polymers and synthetic bioinspired materials holds great promise, since natural bone is a composite structure consisting of carbonated hydroxyapatite (HAp) nanocrystals, collagen, and non-collagenous proteins [4]. 

The architecture of the scaffolds should present high degree of porosity with an interconnected porous network to allow for effective cells, oxygen, nutrient and waste diffusion. Scaffold pore size also significantly affects cellular adhesion and growth, since scaffolds surface area increases as the pore size decreases, thus enhancing cells binding and cell–scaffold interactions. However, the ability of cells to migrate throughout the scaffolds should not be restricted due to the small pore size [5]. As it has been proved, an ideal bone scaffold, should possess bimodal pore size distribution including both macroporosity, with pore size ranging from 100 to 200 µm [6] and microporosity with pore size less than 10 µm [7,8] to improve osteogenesis and angiogenesis in vivo. In addition, mechanical properties, such as compressive strength, elastic modulus, and fatigue strength, comparable to those of natural tissue, significantly affect bone tissue growth and provide the required structural integrity in vivo. However, increased porosity and mechanical stiffness are directly conflicting physical properties and many research efforts [9,10] have focused on methods to improve mechanical behavior of bone scaffolds while retaining an open porous structure. As it has been proved, an ideal bone scaffold should possess a compressive strength comparable to trabecular bone, which is approximately 2–20 MPa [11,12,13] and elastic modulus (E) ranging between 0.1 and 2 GPa, while presenting high porosity, ranging between 50% and 90%, also comparable with the values of cancellous bone. However, cancellous tissue is of anisotropic nature meaning its mechanical properties vary according to the direction in which they are examined [14]. The complexity of the relationship between the microarchitecture and nanoindentation properties of nano-hydroxyapatite/collagen scaffolds, prepared using different approaches, has been previously reported. The nanoindentation Young’s modulus and hardness (H) of these composite materials seem to achieve maximum values for 45–60% HAp content by weight (starting from 200 and 10 MPa, respectively) [15].

3D scaffolds with porous structure can be produced by various methods [16,17]; however, in order to fabricate porous complex-shaped structures with greater precision a lyophilization process can be followed [18,19]. The internal structure and thus the properties of the final scaffold are determined by the suspension parameters (crystal size, suspension stability) and the freezing conditions (temperature and direction of freezing) [20], whereas by controlling ice crystal formation, preferential orientation of the porosity can be achieved leading to anisotropic microstructure [20,21]. Further, it has been illustrated that freeze-drying can provide tailoring at two levels of a hierarchical porous structure consisting of a bimodal pore size distribution of small pores coexisting with larger ones [22,23,24]. 

**Figure 1:**
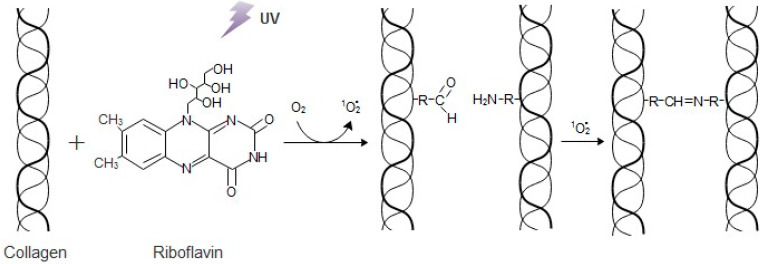
The interaction between collagen and riboflavin under UV irradiation.

In this study, a way to improve the mechanical behavior and stability of bioinspired 3D freeze-dried bone scaffolds, while retaining an internal interconnected porous network, was examined by performing physical cross-linking. To create these 3D scaffolds, a hybrid suspension has been synthesized via a bioinspired approach, based on previous results [25], in which HAp nanocrystals are formed in the presence of collagen fibers and the amino acid **l**-arginine, to mimic the natural process of bone formation. The resulting material presents a bone-like composition and nanostructure, thus its biological properties, in terms of cytocompatibility and tissue regeneration are highly promising. To reinforce the mechanical properties of the produced material, a physical crosslinking method through riboflavin irradiation was performed, since riboflavin is typically used for increasing collagen stiffness [26,27]. Riboflavin (vitamin B_2_), a photosensitive non-toxic compound, reaches absorption peaks at 266 nm, 373 nm, and 445 nm, meaning that it can be activated under UVA or visible blue light lamps. Upon activation, it passes from the ground state to a spin-allowed triple-excited state (Figure 1). The excitation energy is transferred to ground state oxygen causing the formation of singlet oxygen (^1^O_2_), a form in which only the spin of the electrons has changed. This oxygen radical interacts with the carbonyl groups, binding them with the amine groups through the formation of covalent bonds [28,29,30,31,32]. 

Thus, in this study the improvement that riboflavin photo cross-linking could cause to the mechanical strength of the produced 3D scaffolds, was evaluated through nanoindentation studies at 37 °C. The internal porosity of these scaffolds, in terms both of pore size and interconnectivity, was evaluated through SEM and micro-CT analysis, while the effective implementation of the biomimetic synthesis of HAp nanocrystals was confirmed through FT-IR and XRD. The nanomechanical analysis in the biomimetic temperature of 37 °C is intriguing, since at this temperature elastic modulus values both for osteons and the interstitial lamellae present a noteworthy decrease of approximately 40% and 57%, respectively, while hardness values present a decrease of 15.6% and 27.4%, respectively. Indeed, it has been reported that the non-calcified collagen molecules in natural bone degenerate at about 43 °C while calcified ones degenerate at about 150 °C [33,34]. Additionally, in temperature higher than this of human body, the triple helices of type I collagen are unstable [35] and easy to collapse, as it is recorded by Leikina et al [36]. Finally, the biological behavior of the produced hybrid scaffolds was assessed by employing cultures of MG63 human osteosarcoma cells for 24 h, 48 h and 72 h. The cell viability was evaluated both through the MTT assay and a double staining process that can be used for discriminating viable and non-viable cells, while cell attachment and morphology in the scaffolds’ surface was monitored through SEM visualization. 

## 2. Materials and Methods

### 2.1. Materials

HAp nanocrystals have been synthesized employing calcium hydroxide, Ca(OH)_2_ and orthophosphoric acid 85% H_3_PO_4_, as sources of calcium and phosphate ions respectively, which were purchased from Sigma–Aldrich (Germany) and used as received. Collagen fibers and l-arginine purchased from Sigma–Aldrich (Germany) and Fisher Scientific (Belgium) respectively. The photo cross-linking agent, riboflavin-5’-phosphate sodium salt dihydrate, was purchased from Alfa Aesar (United States). MG63 human osteoblast-like cells were purchased from American Type Culture Collection (LGC, EU, Germany). For cell studies, Dulbecco’s Modified Eagle Medium (DMEM), Fetal Bovine Serum (FBS), l-glutamine and the antibiotics (penicillin-streptomycin solution) were all purchased from Gibco, Life Technologies, UK. Cell viability assessment was performed employing Fluorescein Diacetate (FDA) and Propidium Iodine (PI) purchased from Sigma-Aldrich, (Germany). Cacodylate buffer solution and osmium tetroxide, employed for the fixation of the scaffolds before SEM Analysis, were purchased from Merck KGaA, (Germany) and Polysciences Inc., PA respectively.

### 2.2. Hybrid Scaffolds Synthesis

The experimental procedure followed for the synthesis of the suspension that was used to form 3D scaffolds, was based on previous work and it combines chemical precipitation with a sol-gel method [25] for the bioinspired synthesis of HAp nanocrystals in the presence of collagen fibers and the amino acid l-arginine. Concisely, the process began with the preparation of solutions of Ca(OH)_2_ and H_3_PO_4_ in distilled water. Collagen fibrils were dispersed in the H_3_PO_4_ solution under intense stirring. The reaction which results in the formation of HAp nanocrystals was performed under the biomimetic conditions of basic pH and temperature at 38 °C, by adding the required quantities of Ca(OH)_2_ and H_3_PO_4_ solutions to achieve a final Ca/P molar ratio 1/1, so that the reaction takes place in excess of acid, instead of the stoichiometric molar ratio Ca/P = 10/6. The presence of l-arginine, in the form of an aqueous solution, during the HAp formation, was used to enhance its crystal growth and tailor the final crystal size [37]. The collagen and arginine contents were estimated so as to achieve a HAp/Col = 70/30 weight ratio and a Ca^2+^:Arg = 1:1 molar ratio in the final suspension, based on previous results [25].

Subsequently, the hybrid suspension of HAp, collagen and arginine was centrifuged at 10,000 rpm for 25 min and the obtained material was essentially a hydrogel of HAp and biomolecules. This hydrogel was then cast into cylindrical molds with diameter at 0.5 cm and additionally centrifuged ta 10,000 rpm to gradually subtract the water content. Hybrid 3D scaffolds with 0.5 cm diameter and length varying from 3 to 4 cm, were finally produced through a freeze-drying process. HAp aqueous suspensions were first frozen at −55 ^ο^C and then the ice crystals were sublimated under vacuum at 0.35 mbar. 

### 2.3. Crosslinking of the Scaffolds

A 0.01% riboflavin solution was prepared by dissolving riboflavin-5’-phospate in distilled water. The cylindrical scaffolds were submerged in the riboflavin solution and remained for 24 h, to ensure that the photosensitizer has permeated the entirety of the scaffold. After 24 h the riboflavin solution was subtracted and the samples were exposed to UVA light for 1 h. After the irradiation, the scaffolds were lyophilized again and stored at room temperature. Upon completion of the lyophilization procedure, the physicochemical properties of the as-obtained scaffolds were examined. 

### 2.4. Characterization Techniques

#### 2.4.1. Fourier Transform-Infrared Spectroscopy, Ft-Ir

The structure of the compounds and the nature of the bonds developed in the hybrid suspension synthesized, were studied through Fourier transform-infrared spectroscopy (FT-IR) using an Agilent, Cary 630 FTIR-ATR spectrometer by employing a sample of the hybrid suspension in powder form. The collected spectra range from 4000 to 400 cm^−1^. The spectra resolution of the instrument is below 2 cm^−1^.

#### 2.4.2. X-ray Diffraction, XRD 

The crystallinity and phase purity of HAp sample were examined through XRD on a Siemens D5000 diffractometer using a copper anode and a graphite monochromator to generate CuKa1 radiation (λ = 1.5405 Å), powered at 40 kV voltage and 40 mA current. The measurement was conducted in a sample of the hybrid suspension in powder form between 20° to 60° with a step of 0.02°/5 s. 

#### 2.4.3. Microcomputed Tomography (µ-CT)

A study on the interior of the scaffolds micro-architecture was implemented through micro-CT. The samples were imaged by micro-CT (Bruker SkyScan 1272). During acquisition, the samples rotated over 180° with a fixed rotation step. At each angular position a shadow (or transmission) image was acquired. The acquisition settings that are presented in Table 1, were applied. 

**Figure 2:**
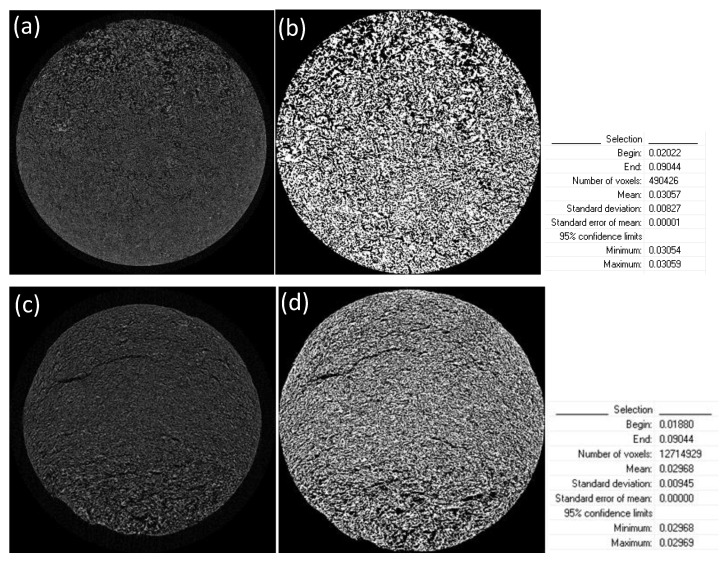
Application of a threshold window from: 0.01880 to 0.09044 AT for scaffolds without crosslinking (**a**) before threshold application and (**b**) after thresholding; and 0.02022 to 0.09044 AT for scaffolds crosslinked with 0.01% riboflavin (**c**) before threshold application and (**d**) after thresholding.

The obtained shadow angular projections were used for the reconstruction of the virtual slices through the samples. Raw data cross sections were generated using NRecon reconstruction software (version 1.7.0.4 by Bruker) by implementing the Feldkamp algorithm. A few reconstruction parameters had to be adjusted manually, to correct reconstruction artifacts (namely misalignment compensation, ring artifact and beam hardening corrections in NRecon software). Automatic thresholding was performed on the reconstructed data to acquire binarized images. The 3D automatic Otsu method was employed. Reconstructed data before and after thresholding are depicted in Figure 2, along with standard deviations and confidence limits of threshold selections. Porosity analysis was conducted with CTAn software. Closed pores are registered as any group of black pixels surrounded by white pixels, while open pores are defined as any space located within a solid object or between solid objects, which has any connection in 3D to the space outside the object or objects. Total porosity is the volume of all open plus closed pores as a percent of the total VOI volume CTAn software manual [38].

#### 2.4.4. Nanomechanical Properties at 37 °C

Mechanical properties of the 3D scaffolds were assessed through nanoindentation test using a Hysitron TriboLab® Nanomechanical Test Instrument. The indentations were performed with a diamond Berkovich tip (three-sided pyramid, elastic modulus 1140 GPa, Poisson ratio 0.07). The instrument allows the use of loads varying from 1 to 10,000 µΝ, while it monitors the depth of indentation as a function of the load applied on the sample. The applied load was set to reach 1 nN and the indentation depth was 0.04 nm. 

#### 2.4.5. Cell Cultures

The methodology for the assessment of the biological properties of the produced scaffolds, has been established according the ISO 10995-3 guidelines and the international literature. Specifically, MG63 human osteoblast-like cells were maintained in Dulbecco’s Modified Eagle’s Medium (DMEM) low glucose, supplemented with 10% fetal bovine serum (FBS), and 1% antibiotics (penicillin–streptomycin solution) in controlled atmosphere (5% CO_2_; T = 37 °C). The cells were used between the 3rd and 4th passages. MG63 cells were seeded onto the scaffolds and to the cell tissue plastic (TCP) as control samples, at the density of 1 × 10^4^ cells/cm^2^. All experiments were run in triplicate in 3 independent runs.

#### 2.4.6. Scanning Electron Microscopy (SEM)

The internal architecture of the scaffolds was observed through micrographs obtained using a Hitachi TM3030Plus Tabletop Scanning Electron Microscope, operated at 5 kV. Analysis was carried out by employing both horizontal and vertical cross sections of the cross-linked and non-cross-linked scaffolds. Moreover, cellular adhesion and distribution within the scaffolds were assessed by using SEM. Cells fixation on both scaffolds and control samples occurred in 3% (*v*/*v*) glutaraldehyde in 0.1 M sodium cacodylate buffer solution (pH 7.4), post-fixed in 1% osmium tetroxide followed by dehydration through careful addition of a series of increasing concentrations of ethanol 30%, 50%, 70%, 80%, 95%, and 100% (*v*/*v*) in dH_2_O for 10 min at 4 °C. Finally, samples were left to air dry in a fume cabinet. Prior to all SEM examination, samples were sputter coated with gold.

#### 2.4.7. MTT Assay

MTT assay was used to assess cell cells viability. MG63 cells were seeded onto the scaffolds in a 48-well plate for 24 h, 48 h, and 72 h. At each experimental point, the medium from cultures was removed; 200 μL of MTT (Sigma-Aldrich Ge) solution and 1.8 mL of DMEM without Phenol red were added to each well; the multi-well plates were incubated at 37 °C for a further 4 h. After discarding the supernatants, the dark blue formazan crystals were dissolved by adding 2 mL of solvent (0.1N HCl in absolute isopropanol) and quantified spectrophotometrically (VersaMax ELISA Microplate Reader, Biocompare, USA) at 570 nm.

#### 2.4.8. FDA/PI Double Staining 

Cell viability on the seeded scaffolds, was assessed by using aa double staining process with propidium iodide and fluorescein diacetate (PI/FDA) (Sigma-Aldrich, Ge) after 24 h, 48 h, and 72 h of culturing. The samples were washed with PBS and stained with FDA under darkness. To prepare the FDA staining solution, 1 mg/mL FDA were dissolved in DMSO and further diluted to a concentration of 3 μg/mL in PBS. To prepare the PI staining, 1 μg/mL PI was added in PBS. After an additional washing step, the samples were observed under a fluorescent microscope (Olympus CKX41, Hamburg, Germany), excited with FTIC and TEXAS RED lasers. Living cells appeared to be green, and the dead ones, red. The percentages of the living cells were determined by using ImageJ program. Five to ten different random areas of each image from the scaffolds and the control samples were considered in order to measure green (alive) and red (dead) cells.

#### 2.4.9. Statistics

Statistical analysis was performed using the GraphPad Prism 8.0 software (San Diego, CA, USA) using Non-Parametric *t*-Test. *p*-values of < 0.05 were considered statistically significant.

## 3. Results and Discussion

### 3.1. Fourier Transform-Infrared Spectroscopy

**Figure 3:**
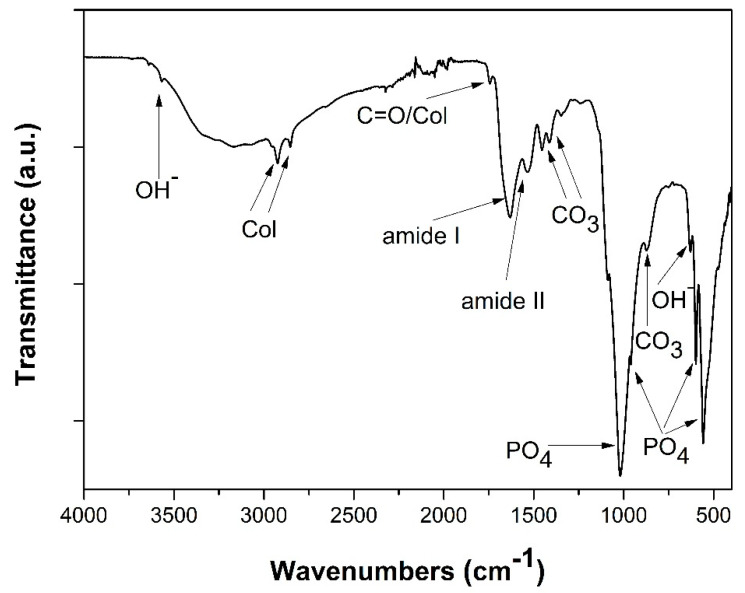
FT-IR spectra of hydroxyapatite (HAp)-Col-Arg samples developed through a bioinspired approach.

FT-IR analysis was used to evaluate the chemical composition of the synthesized hybrid suspension. All the characteristic bands of the interactions between the organic and inorganic compounds involved and formed were ascertained in the spectrum of Figure 3. Initially, the characteristic bands of the PO_4_^3−^ groups can be easily distinct proving the formation of HAp crystals. Specifically, the bands noted at 556 and 596 cm^−1^ correspond to the v_4_ bending modes of O-P-O bond [39,40]. The band at 1016 cm^−1^ arises from the anti-symmetric v_3_ stretching modes of P-O bonds, while the band at 963 cm^−1^ can be assigned to the symmetric v_1_ stretching mode of the phosphate groups (ΡO_4_) [38,40]. Furthermore, the symmetric stretching mode of OH^−^ ions is attributed to the 3644 cm^−1^ band, along with the 645 cm^−1^ band representing the librational mode of hydroxyl groups [41,42,43,44,45].

In addition, the characteristic bands of collagen molecules interactions at 2930 and 2853 cm^−1^ are present in the same spectra. The band representing amide I appears at 1627 cm^−1^, whilst for amide II the band is traced at 1546 cm^−1^. Another band proving the presence of collagen in the scaffolds is at 1748 cm^−1^, due to the stretching vibrations of carbonyl groups (C=O), situated at the amide I part of collagen [46,47,48]. 

Finally, the bands at 1453, 1408, 1412, and 871 cm^−1^ prove the existence of carbonate ions in the HAp scaffolds, possibly detected due to the presence of an essential quantity of organic compounds and/or traces of dissolved CO_2_. The band at 1453 cm^−1^ attributes to the v_3_ stretching vibrations of CO_3_^2^, together with the band appearing at 875 cm^−1^ representing the v_2_ stretching modes of said groups [45,46,49]. Therefore, the formation of HAp crystals through the biomimetic synthesis that was performed, was confirmed, in complete agreement with the equivalent results of the previous works upon which this study is based. 

### 3.2. X-Ray Diffraction Analysis

**Figure 4:**
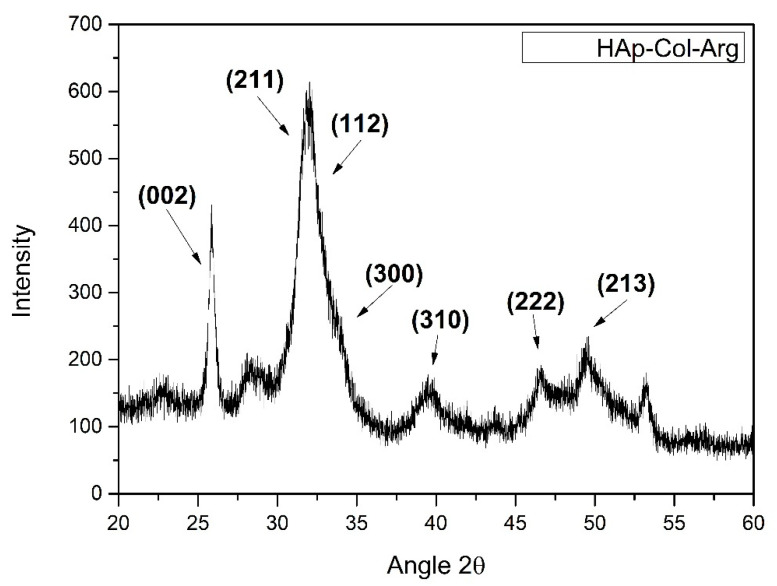
XRD pattern for HAp-Col-Arg samples developed through a bioinspired approach.

X-ray diffraction analysis provided information about the crystallinity and the phase purity of synthesized HAp crystals. Phase identification was carried out by comparing the diffraction peaks of the obtained samples with those of pure hydroxyapatite (JCPDS 09-0432). Figure 4 spectrum showcases all the characteristic crystal lattices of HAp. The diffraction peaks that appear coincide with the characteristic peaks of hexagonal hydroxyapatite. It is indeed noted that there are no precursors phases of HAp detected, nor other calcium phosphate salts are present in the hybrid material produced. 

It is noteworthy to mention that the presence of the biopolymers during synthesis of HAp crystals is crucial. Collagen fibrils provide suitable sites for HAp nucleation, since collagen’s structure allows for the formation of binding positions. One of the three amino acid chains of collagen, ends with an amine group which does not form a bond with the carboxylic group of the following chain, thus creating the appropriate binding sites [50,51]. These spaces cause chelation between collagen and calcium ions (Ca^2+^), with the binding sites of collagen acting as the polydentate ligand and the calcium ions as the central atom.

Besides collagen, nature uses small quantities of various biomolecules to control the development of HAp crystals, such as amino acids and non-collagenous proteins. In this study, the amino acid l-arginine that was used, provides a guanidinium group on one side, capable of binding phosphate ions (PO_4_^2−^) and a carboxyl group on the other side, capable of binding calcium ions (Ca^2+^). Therefore, this amino acid enhances the formation of apatite crystals while as it has been proved l-arginine can also regulate the final HAp crystal size [52,53,54]. In fact, it offers the possibility of tailoring the hydroxyapatite’s crystal morphology as it limits the growth of hydroxyapatite crystals along the a and b directions, while promoting the growth along the c-axis [55].

### 3.3. Scanning Electron Microscopy Analysis

**Figure 5:**
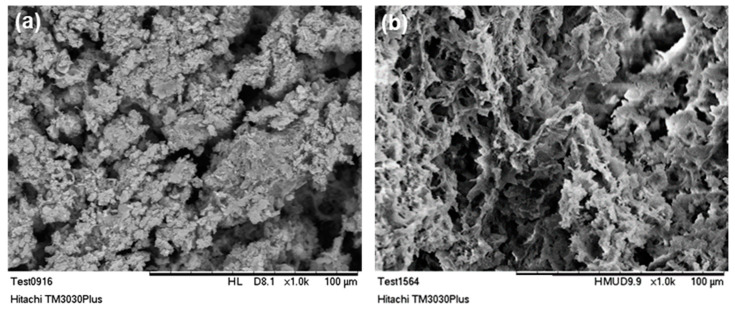
SEM micrographs of 3D hybrid scaffolds (**a**) without crosslinking and (**b**) crosslinked with 0.01% riboflavin.

SEM micrographs, presented in Figure 5, revealed that the lyophilization of the composite hydroxyapatite suspensions, led to three-dimensional hybrid scaffolds with open porosity, mimicking composition and structure of the extracellular matrix of natural bone tissue [56,57]. By comparing the porous structure prior and after the photo cross-linking process, it can be stated that the internal micro-architecture of the scaffolds is altered, and a bimodal pore size distribution has been created. The porous system is consisted of micropores with size from 10 up to 25 µm, and macropores with a mean size around 100 µm. A similar porous structure was revealed in various cross and vertical sections examined (data not shown), thus it is estimated that the pores are in fact interconnected and the morphology of the crosslinked scaffold resembles natural bone structure [58]. 

As it is already mentioned, the existence of an interconnected porous network is of vital importance for scaffolds targeting bone tissue regeneration, since it is essential for the unhindered circulation of cells, nutrients and cellular waste products. Moreover, the bimodal pore size distribution, can be favorable for both cell and blood capillary growth [59,60]. Indeed, micropores (ranging from 5–20 µm) may contribute to the circulation of body fluids, while macropores (>100 µm) are responsible for creating a suitable environment for cell distribution, adhesion, proliferation and subsequent vascularization [61,62,63,64].

### 3.4. Micro-Computed Tomography (µ-CT)

The inner microarchitecture of the scaffolds was examined by micro-CT scanning. Micro-CT is a fast, non-destructive technique and is this case it provides information about the interconnectivity of the pores existing in the scaffold. The cross-sections acquired after the scanning get reassembled in order to create a 3D model of the specimen. Analysis was conducted at three representative regions of each specimen.

**Figure 6:**
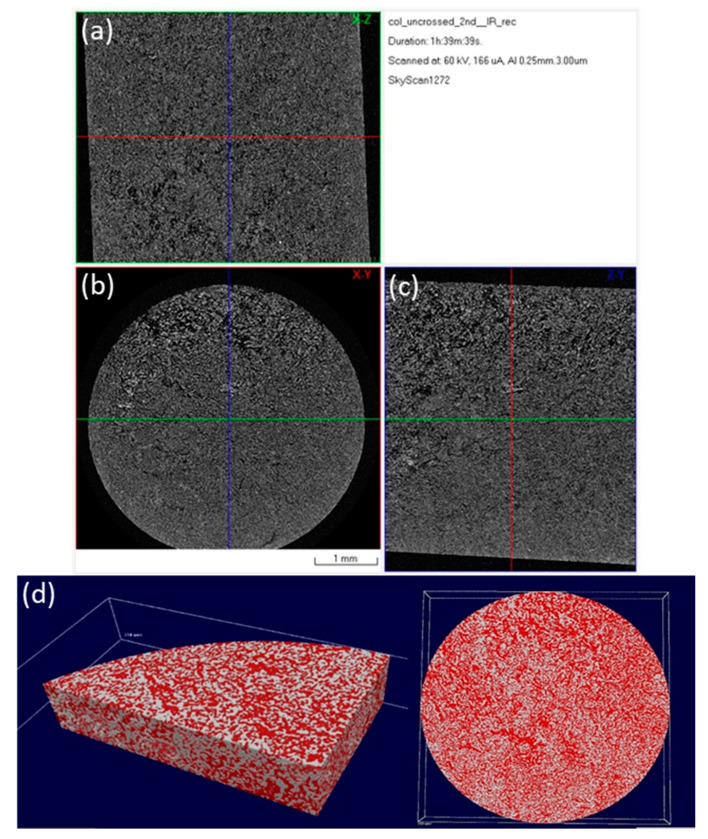
Micro-CT images of a 3D hybrid scaffold without crosslinking: reconstructed results visualized as a set of orthogonal slices crossed at selected points (**a**) XZ cross section, (**b**) XY cross section (perpendicular to Z axis of the specimen), (**c**) ZY cross section; (**d**) 3D surface rendered model from micro-CT scan presenting internal porosity (red) in specimen volume (white).

**Figure 7:**
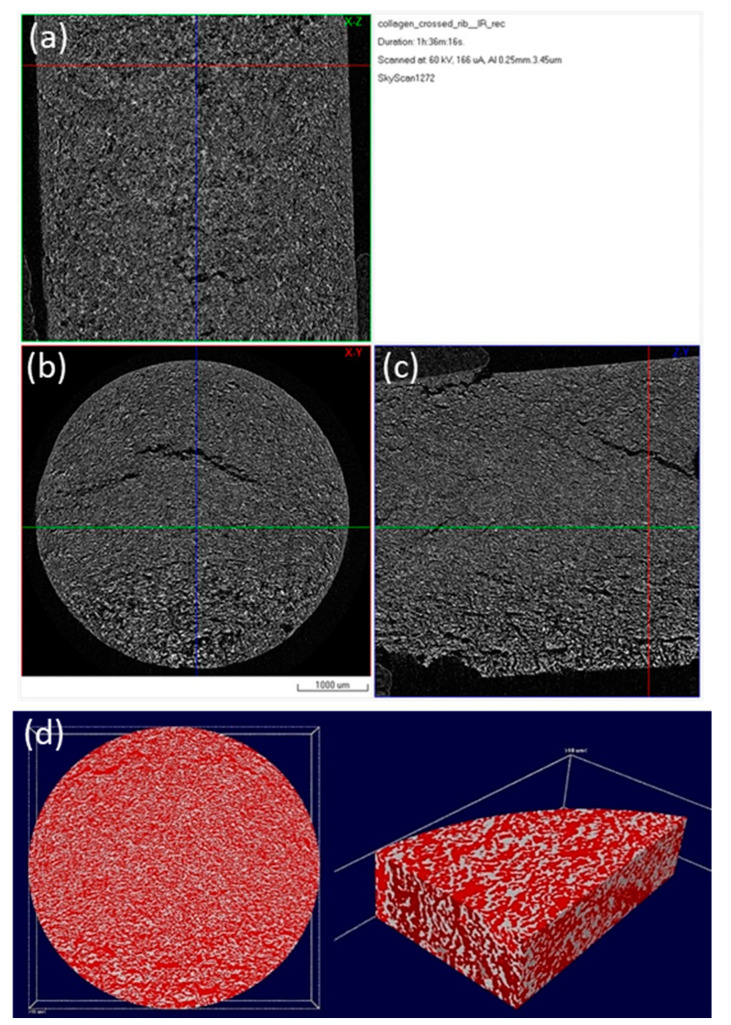
Micro-CT images of a 3D hybrid scaffold crosslinked with 0.01% riboflavin: Reconstructed results visualized as a set of orthogonal slices crossed at selected points (**a**) XZ cross section, (**b**) XY cross section (perpendicular to Z axis of the specimen), (**c**) ZY cross section, (**d**) 3D surface rendered model from micro-CT scan presenting internal porosity (red) in specimen volume (white).

The images presented in Figure 6 and Figure 7, represent a cross-section of the reconstructed sample volume. Some of the cross-sections depicted are perpendicular to the Z axis (longitudinal) of the specimens while some others are parallel to the same axis (Figure 6). A few indicative 3D images are also illustrated in Figure 7. The light-colored points in these images, represent the main skeleton of the scaffold, whilst the dark parts represent the pores. The reconstruction of all the cross-sections together results in a 3D surface model which allows for the observation of the inner “channels” running through the scaffold. The received models confirm the existence of an interconnected porous network in the interior of the scaffolds, also supported by porosity analysis carried out with CTAn software. 

**Figure 8:**
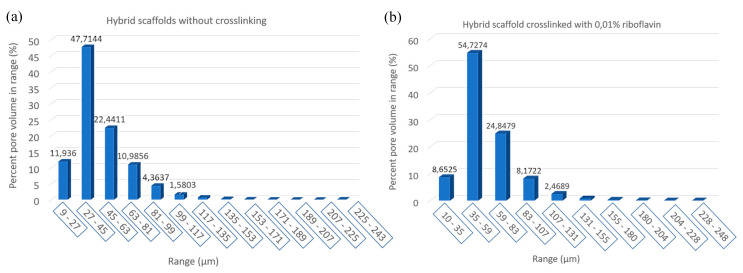
Block charts of pore size distribution in the produced scaffolds: (**a**) Without crosslinking and (**b**) crosslinked with 0.01% riboflavin.

After photo crosslinking with riboflavin, a slight increase to the porosity of the scaffold was observed in accordance with the SEM micrographs. This can be attributed to the fact that cross-linking (either physical or chemical) affects the scaffold’s pore size, since the shrinkage of the hydrogel structure is inhibited due to a rearrangement of collagen chains [65,66]. The porosity of the non-crosslinked scaffold was estimated to be 54%, a property that reached 61% after the cross-linking process (Table 2). This porosity value is within the porosity range that is required for a scaffold to be used in bone tissue regeneration applications [12]. The pore size seems unchanged between the two scaffolds with micropores ranging between 15–25 µm and macropores appearing at around 100 µm (Figure 8), in full agreement with the results obtained from SEM analysis. 

### 3.5. Nanomechanical Properties at 37 °C

The adhesion protocol used to examine the nanomechanical properties of the materials includes the use of a Berkovich tip, approaching the sample at 500 nm above its surface. Subsequently, the tip penetrates the specimen at a predetermined depth (1000 nm), thus performing the loading cycle. Unloading follows, carried out at the same rate. All the measurements were held in the same experimental conditions of 45% humidity and 37 °C. An average of 10 measurements, taken at a 50 µm distance from each other, was kept for statistical reasons. The aim of this test was to observe the alternation of the material’s mechanical behavior, after it had been subjected to photo crosslinking.

**Figure 9:**
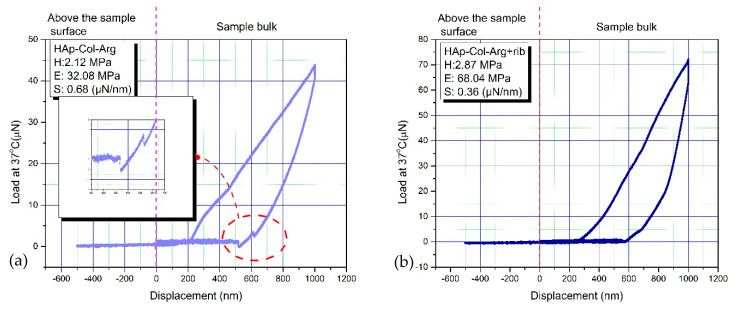
A load-displacement curve for a 3D hybrid scaffold: (**a**) without crosslinking and (**b**) crosslinked with 0.01% riboflavin.

The elastic modulus(E), stiffness(S) and hardness(H) of the specimens were calculated, based on the load-displacement curves, using to the Oliver-Pharr model. The resulting values were: H = 2.12 MPa and Ε = 32.08 MPa for the material without any crosslinking, whilst after crosslinking, these properties increased to reach the values: H = 2.87 MPa and Ε = 68.04 MPa. The values of stiffness are also depicted in the graphs of Figure 9. The stiffness was found to be S = 0.36 µN/nm and S = 0.68 µN/nm, for the non-crosslinked and crosslinked scaffold respectively. Through nanoindentation, it is reported that higher mechanical properties, especially for the elastic modulus, are achieved, in crosslinked samples than for the un-crosslinked ones, in accordance with relevant research studies [67]. These values are in the range of the values of hardness and elastic modulus that have been recorded for cancellous bone (hardness: 2–20 MPa and elastic modulus: 0.1–2 GPa) [11,12,13,14], thus proving that the physical cross-linking process followed led to promising results regarding the mechanical behavior of the produced scaffolds, as candidate material for bone tissue regeneration. Pure collagen is reported to show an average tensile strength of 1.68 ± 0.10 MPa and modulus of 6.21 ± 0.8 MPa with an average strain value of 55 ± 10%. As HAp content increases, the ultimate strength increases to 5 ± 0.5 MPa and modulus to 230 ± 30 MPa. The increase in tensile modulus may be attributed to an increase in rigidity over the pure polymer when the HAp is added and/or the resulting strong adhesion between the two materials [64]. Nevertheless, from these graphs it is apparent that the examined scaffolds possess an elastic surface area (0–250 nm) in both cases, exhibiting significantly low resistance to the imposed load. A substantial adhesion is observed in the curve deriving from the non-crosslinked scaffold, highlighted in the magnified part of the chart (Figure 9a). This phenomenon decreases considerably when crosslinking is introduced to the hybrid scaffold (Figure 9b). The elastic deformation is higher in the case of the crosslinked sample, a remark based on the proportion of surface included in the load-displacement curve. 

### 3.6. Cell Viability Studies

#### 3.6.1. MTT Assay

**Figure 10:**
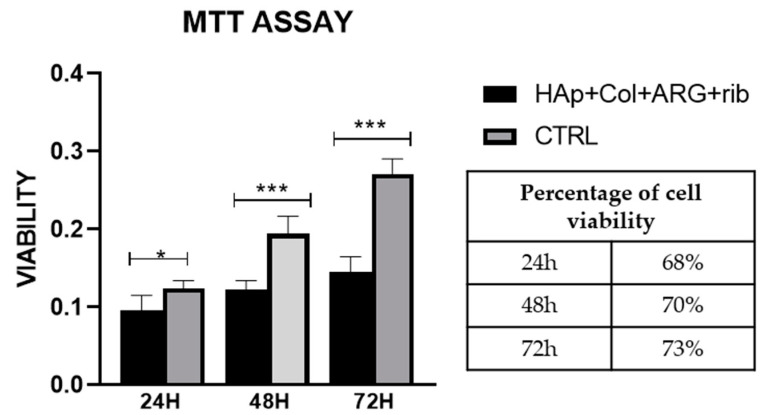
MTT assay on HAp-Col-Arg scaffolds crosslinked with 0.01% riboflavin. Comparison is made between the scaffolds and the control sample (cells seeded on cell tissue plastic (TCP)). Percentage of viability corresponds to the ratio of the sample in respect to the control at each end point.

The effect of the scaffolds surface morphology and chemistry on cells proliferation rate is reported in Figure 10. Premature osteoblasts displayed statistically significant increase in their proliferation rate after 24 h, 48 h, and 72 h seeding on the hybrid scaffolds, which is depicted as an increase in the % absorbance levels. Regardless the higher % absorbance levels of the control samples, (which may also lead to confusing results), the % viability values of the cells onto the scaffolds, over time, are quite promising. These results indicate a clear tendency of cells to proliferate on the scaffolds, thus proving that in terms of cell viability, the produced hybrid scaffolds present excellent behavior. 

#### 3.6.2. FDA/PI Double Staining 

**Figure 11:**
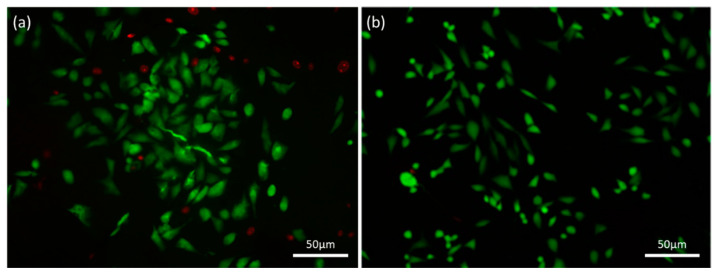
FDA/PI staining on (**a**) a HAp-Col-Arg scaffold crosslinked with 0.01% riboflavin (184 cells in total and 32 of them are red) in comparison with (**b**) the control sample (225 cells in total and 2 of them are red). Both samples were stained after 72 h of cell culture.

The viability of the cells seeded on the scaffolds was determined employing a simultaneous double-staining procedure. FDA and PI were used to discriminate living (green) and non-living cells (red). As it is revealed from the Fluorescence Microscope pictures (Figure 11), hybrid scaffolds’ surface offers a cell friendly environment permitting high levels of viability and a low rate of apoptosis. The population of the green/alive cells measured in the scaffolds crosslinked with 0.01% riboflavin is greater than that of the red/dead cells in all endpoints examined. Specifically, in Figure 11a from the 184 cells that were counted, only 32 of them were dead (red), while in the control sample (Figure 11b), after 72 h of culture, from the 225 cells counted only 2 of them were dead. The osteoblasts seeded on the scaffold tend to populate the whole volume of the scaffold (in all directions); however, cell cultures were performed in static conditions, and hence infiltrating in-depth in the cavernous porous, may have caused cell death due to the lack of oxygen and nutrients. 

#### 3.6.3. SEM Analysis—Cell Morphology Evaluation

**Figure 12:**
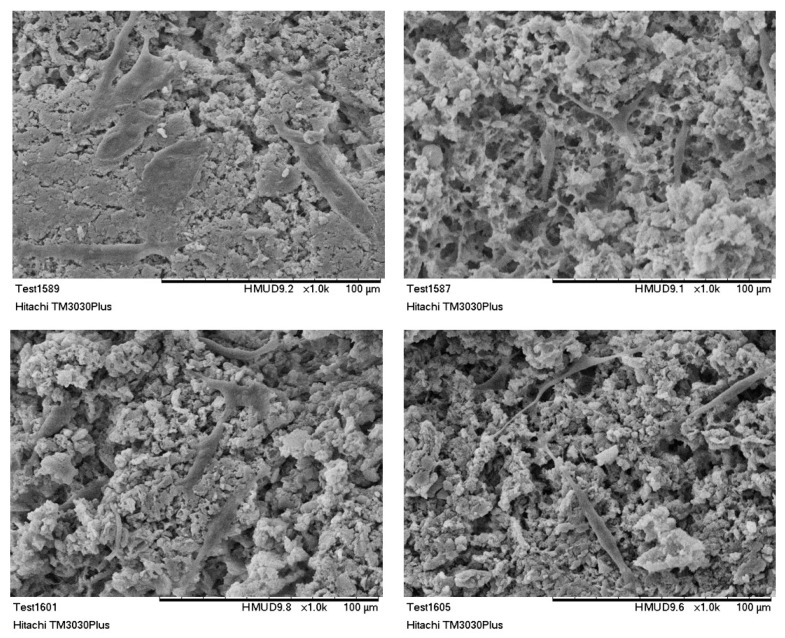
SEM images of MG63 cells cultured for 72 h on HAp-Col-Arg scaffolds crosslinked with 0.01% riboflavin.

SEM analysis was employed to monitor cells’ attachment and morphology on the hybrid scaffolds [68,69,70]. The produced scaffolds supported the attachment and in vitro proliferation of the cultured osteoblasts. Seventy-two hours after seeding, the osteoblasts appear to be distributed evenly over the scaffold surface. As it is presented from Figure 12, MG63 cells maintained their tissue specific morphology. Cells were found to be well spread, enabling interactions both among them and with the scaffold’s surface. SEM micrographs confirmed the results obtained both by the MTT assay and the double staining process. Additionally, as it was also revealed from the FDA/PI staining process, cells penetrated the scaffold and were able to populate the scaffold at different depths, proving that the as developed bimodal pore size distribution was favorable for cells’ attachment, ingrowth, and proliferation. 

## 4. Conclusions

Within this study, a photo crosslinking method, employing 0.01% riboflavin solution, was followed for the enhancement of the mechanical properties of a hybrid 3D porous scaffold, fabricated through lyophilization by employing a suspension of HAp nanocrystals synthesized in the presence of collagen type I fibers and the synergistic action of the amino acid l-arginine. The formation of HAp nanocrystals with bone-like morphology, through the biomimetic approach followed, was confirmed via FT-IR and XRD analysis, as phase purity, crystallinity and absence of any precursor phase have been proved. Therefore, ensuring the cytocompatibility of the final scaffolds as well as providing a promising environment for osteogenesis. Since, the afore-mentioned scaffolds target bone tissue regeneration, it was deemed necessary to evaluate their internal porosity in combination with their mechanical behavior while also assess their biocompatibility.

The physical cross-linking process described successfully improved the mechanical properties of the produced scaffolds. Indeed, the elastic modulus and hardness values of the crosslinked scaffolds increased, compared to the non-crosslinked ones, and the values achieved are in the range of the values that have been recorded for natural trabecular bone. Especially, it is noteworthy that the elastic modulus values were more than doubled after the physical cross-linking process with riboflavin, reaching E = 68.04 MPa, thus proving the potential for this type of scaffolds’ mechanical reinforcement. 

Additionally, as it has been proved from the SEM analysis, lyophilization process resulted in scaffolds with an open porous complex architecture with highly interconnected pores, mimicking the structure of the extracellular matrix of natural bone tissue. Specifically, these structures demonstrated bimodal pore size distribution, with a mean value at 100 µm for the macroporosity and a range between 10-20 µm for the smaller ones (microporosity). This pores size distribution is expected to be favorable for cell attachment, nutrient and waste diffusion as well as for vascularization and new bone tissue formation, while bimodal pore size distribution is expected to further enhance cell growth. The development of an interconnected porous network and the pore size distribution were also confirmed through micro-CT analysis. The increase in the porosity of the scaffolds from 54% up to 61% after photo crosslinking with riboflavin, was also depicted through µ-CT scanning, as a result of collagen chains rearrangement leading to inhibition of hydrogel’s shrinkage. The evaluation of the biological behavior of these scaffolds proved that cell viability, seeded on the hybrid scaffolds, exhibited a progressive trend during the second and third day of culturing. By comparing the MTT assay results with those obtained from the FDA/PI viability test, it is clearly suggested that the scaffolds’ materials present cytocompatibility and provide a cells friendly environment. Cells visualization through SEM analysis, revealed that the osteoblasts cultured populated the scaffolds without losing their typical morphology, they were well spread, while the interactions both among them and with the scaffold’s surface were enhanced. These results clearly suggested that cells increased their population and maintained high levels of viability in all time intervals examined. The suitability of these scaffolds to support cells’ attachment and enhance their proliferation can be attributed to the presence both of collagen and calcium phosphate salts.

These results indicate that these scaffolds are promising materials to be used in the field of tissue regeneration and further investigations should be carried out in order to optimize the interconnectivity of their internal porous network. Additionally, the as produced scaffolds could be assessed in long-term cultures to gain insights regarding their osteogenic differentiation and their osteoinductive properties.

## Figures and Tables

**Table 1:** micro-CT acquisition settings.

Filter = Al 0.25 mm Source Voltage (kV) = 60 Source Current (uA) = 166 Image Pixel Size (um) = 3.00, 3.45 Camera binning = 2 × 2	Reference Intensity = 57,000 Exposure (ms) = 2005 Rotation Step (deg) = 0.200 Random Movement = ON (10) Flat Field Correction = ON

**Table 2:** Calculations of closed, open and total porosity generated by CTAn software.

Scaffolds Without Crosslinking	Scaffolds Crosslinked with 0.01% Riboflavin
Closed porosity: 0.011% Open porosity: 54.13% Total porosity: 54.13% Standard deviation of structure separation: 7.13 µm	Closed porosity: 0.002% Open porosity: 61.42% Total porosity: 61.42% Standard deviation of structure separation: 6.61 µm
